# Mapping Brain Lesions to Conduction Delays: The Next Step for Personalized Brain Models in Multiple Sclerosis

**DOI:** 10.1002/hbm.70219

**Published:** 2025-05-03

**Authors:** C. Mazzara, A. Ziaeemehr, E. Troisi Lopez, L. Cipriano, M. Angiolelli, M. Sparaco, M. Quarantelli, C. Granata, G. Sorrentino, M. Hashemi, V. Jirsa, P. Sorrentino

**Affiliations:** ^1^ Department of Promoting Health, Maternal‐Infant. Excellence and Internal and Specialized Medicine (PROMISE) G. D'alessandro University of Palermo Palermo Italy; ^2^ Institute of Biophysics, National Research Council Palermo Italy; ^3^ Inst Neurosci Syst Aix Marseille Univ, INSERM, INS Marseille France; ^4^ National Research Council Institute of Applied Sciences and Intelligent Systems Pozzuoli Italy; ^5^ Department of Medical Motor and Wellness Sciences University of Naples “Parthenope” Naples Italy; ^6^ Unit of Nonlinear Physics and Mathematical Models, Department of Engineering Campus Bio‐Medico University of Rome Rome Italy; ^7^ Department of Advanced Medical and Surgical Sciences University of Campania Luigi Vanvitelli Caserta Italy; ^8^ Biostructure and Bioimaging Institute, National Research Council Naples Italy; ^9^ Department of Economic, Legal, Informatics and Motor Sciences University of Naples Parthenope Nola Italy; ^10^ ICS Maugeri Hermitage Napoli Napoli Italy; ^11^ University of Sassari Department of Biomedical Sciences Sassari Italy

**Keywords:** Bayesian inference, conduction delays, magnetoencephalography, multiple sclerosis, personalized brain models

## Abstract

Multiple sclerosis (MS) is a clinically heterogeneous, multifactorial autoimmune disorder affecting the central nervous system. Structural damage to the myelin sheath, resulting in the consequent slowing of the conduction velocities, is a key pathophysiological mechanism. In fact, the conduction velocities are closely related to the degree of myelination, with thicker myelin sheaths associated to higher conduction velocities. However, how the intensity of the structural lesions of the myelin translates to slowing of nerve conduction delays is not known. In this work, we use large‐scale brain models and Bayesian model inversion to estimate how myelin lesions translate to longer conduction delays across the damaged tracts. A cohort of 38 subjects (20 healthy and 18 with MS) underwent MEG recordings during an eyes‐closed resting‐state condition, along with MRI acquisitions and detailed white matter tractography analysis. We observed that MS patients consistently showed decreased power within the alpha frequency band (8–13 Hz) as compared to the healthy group. We also derived a lesion matrix indicating the percentage of lesions for each tract in every patient. Using large‐scale brain modeling, the neural activity of each region was represented as a Stuart‐Landau oscillator operating in a regime showing damped oscillations, and the regions were coupled according to subject‐specific connectomes. We propose a linear formulation to the relationship between the conduction delays and the amount of structural damage in each white matter tract. Dependent upon the parameter γ, this function translates lesions into edge‐specific conduction delays (leading to shifts in the power spectra). Using deep neural density estimators, we found that the estimation of γ showed a strong correlation with the alpha peak in MEG recordings. The most probable inferred γ for each subject is inversely proportional to the observed peaks, while power peaks themselves do not correlate with total lesion volume. Furthermore, the estimated parameters were predictive (cross‐sectionally) of individual clinical disability. This study represents the initial exploration showcasing the location‐specific impact of myelin lesions on conduction delays, thereby enhancing the customization of models for individuals with multiple sclerosis.

## Graphical abstract

This study examines how myelin lesions in multiple sclerosis (MS) affect nerve conduction delays using a whole‐brain model and Bayesian inversion. The strength of lesion‐induced delays (parameter gamma) correlates with alpha peak shifts in MEG recordings, but not with total lesion load, and predicts clinical disability.

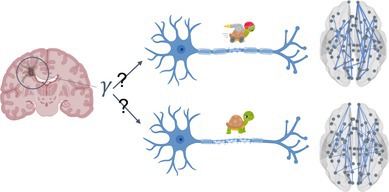

## 1 Introduction

Multiple sclerosis (MS) is a chronic autoimmune disease that affects the central nervous system, provoking lesions that result in a wide variety of physical and cognitive disabilities, including paresthesia, vision loss, ataxia, mental changes, and weakness (Gaby 2013; Ghasemi et al. 2017). The etiology of MS is still unclear; nonetheless, it is understood that it is multifactorial and consists of complex interactions among different genetic and environmental factors (Bernard and de Rosbo 1992; Filippi et al. 2018; Ghasemi et al. 2017; Yamout and Alroughani 2018). The pathogenesis of MS entails a cascade of events in the immune system which result in inflammation, nerve demyelination, axonal/neuronal damage, and death of neuronal cells (Ghasemi et al. 2017; Miller 2012).

Attacks on the myelin play a pivotal role in MS pathophysiology. Damage to the myelin, such as demyelination or decompaction, can lead to impaired conduction with lower velocities (Fowler and Gilliatt 1981; Gutiérrez et al. 1995; Reutskiy et al. 2003). In fact, the conduction velocity of action potentials along the white matter bundles is proportional to the degree of myelination (Gutiérrez et al. 1995). Therefore, the thickness of the myelin sheath, which is also closely associated with the axon diameter, plays a significant role in determining conduction velocity (Gillespie and Stein 1983; Ritchie 1982; Sanders and Whitteridge 1946).

Along these lines, characterizing conduction velocity and delays might be an effective way to estimate subtle damage to the myelin that, while contributing to the clinical picture, might not yet manifest as overt structural changes and, hence, might not be detected by structural imaging alone. Evoked potentials have been used to measure conduction velocity on selected white matter tracts, but assessing the delay across the whole brain is not feasible (Leocani et al. 2000). However, the overall dynamics, which depend on all the delays, are clinically relevant (Sorrentino et al. 2022). Recent studies have taken a stride forward in evaluating conduction delays and velocity through structural magnetic resonance imaging (MRI) (Drakesmith et al. 2019; Mancini et al. 2021), providing a non‐invasive alternative. Mancini (Mancini et al. 2021) and Drakesmith (Drakesmith et al. 2019) leveraged MRI‐derived microstructural measures to estimate conduction velocity and delays distributions across the whole brain, using features such as the axonal diameter and the myelin content (calculated through the g‐ratio). However, there are still limitations, such as the MRI resolution limit, which can affect the ability to accurately measure microstructural parameters, hindering, for example, the complete characterization of axonal diameter distribution and, consequently, leading to estimation errors, especially for smaller axons. Crucially, how exactly myelination, as estimated by the g‐ratio, maps onto the conduction velocities remains unknown. One might expect that the relation between the g‐ratio and the actual conduction velocities might change as a function of the edge considered. However, the validation of MRI measures is limited (Mancini et al. 2021). Furthermore, accurate estimates of conduction velocities are generally not obtainable in scenarios characterized by low axon density (Drakesmith et al. 2019).

Deriving the average conduction velocity across the entire brain poses challenges mainly due to the lack of efficient model inversion algorithms operating at such a large scale (Hashemi et al. 2020, 2021). To overcome this difficulty, (Sorrentino et al. 2023) opted to infer patient‐specific average conduction velocities by integrating diffusion tensor imaging and source‐reconstructed magnetoencephalography (MEG) data into a personalized large‐scale brain model. Such personalized models were then inverted using a simulation‐based inference (SBI) scheme with a class of deep neural networks known as neural density estimators (Gonçalves et al. 2020; Hashemi et al. 2023; Liu et al. 2021; Ziaeemehr et al. 2025) to estimate the most likely conduction velocities.

**FIGURE 1:**
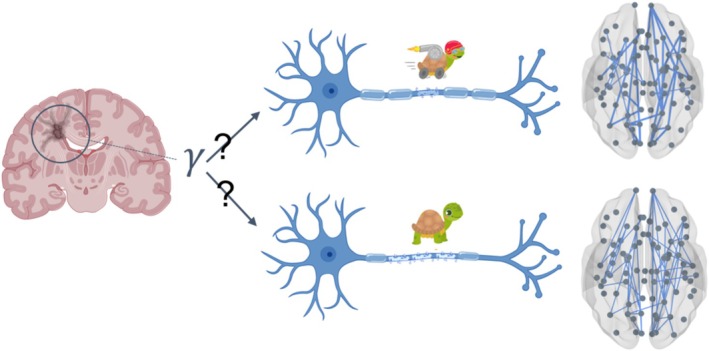
Model Overview: Gamma Parameter. On the left, the diagram illustrates a typical lesion in multiple sclerosis (MS). The variable gamma (γ) in our model represents the relationship between the structural lesion and the corresponding slowing of the velocity in the propagation of the nervous signal. The value of gamma gauges the severity of the effect of the lesion. Although the signal transmission is slower in patients as compared to the controls, the slowing can vary greatly. At the top of the diagram, less severe MS is depicted, characterized by fewer lesions that translate less prominently to higher delays, as represented by the thicker connections between the nodes. At the bottom, a more severe condition is represented, with more myelin lesions, severely slowed conduction velocities, and, in the whole brain model, thinner connections.

In this paper, we hypothesize that a specific relationship exists between the extent of the myelin lesions, as measured from structural imaging, and the consequent rise in the conduction delays across the brain edges (Figure 1). To test for the existence of such a relationship, and to invert it at the patient‐specific level, we used the neural density estimators in each participant at the whole‐brain level.

As explained in more detail in Sorrentino et al. (2023), each subject underwent both MEG—recorded during an eyes‐closed resting‐state condition—and MRI, including white matter tractography analysis. Additionally, we obtained a lesion matrix describing the percentage of lesion for each edge in each patient. We used a large‐scale brain models where each region of interest (ROI) is represented as a Stuart‐Landau oscillator in a regime with damped oscillations. For each subject, we utilized the shift in the power spectrum to quantify the effect of the structural damage, measured in each white‐matter bundle, on the conduction delays across these edges.

## 2 Results

In this study, we used SBI with a neural network‐based approach to estimate the parameters of whole‐brain models, given on Stuart‐Landau oscillators, in patients with MS and controls. The model was used to train SBI and then infer patient‐specific model parameters from empirical MEG recordings. Specifically, we applied neural posterior estimation (NPE), where a deep neural network approximates the posterior distribution of model parameters given MEG observations. This is the state‐of‐the‐art conditional neural density estimator to estimate posterior distribution as outputs, from low‐dimensional MEG features (e.g., power spectral density, peak amplitude, peak frequency) as network inputs. The architecture followed the default SBI toolbox implementation (Tejero‐Cantero et al. 2020). To train the network, we generated synthetic MEG data by simulating the Stuart‐Landau model given random parameters taken from prior distribution. The network is trained by minimizing the negative log‐likelihood and validated on separate synthetic data by comparing inferred posteriors to ground truth values.

**FIGURE 2:**
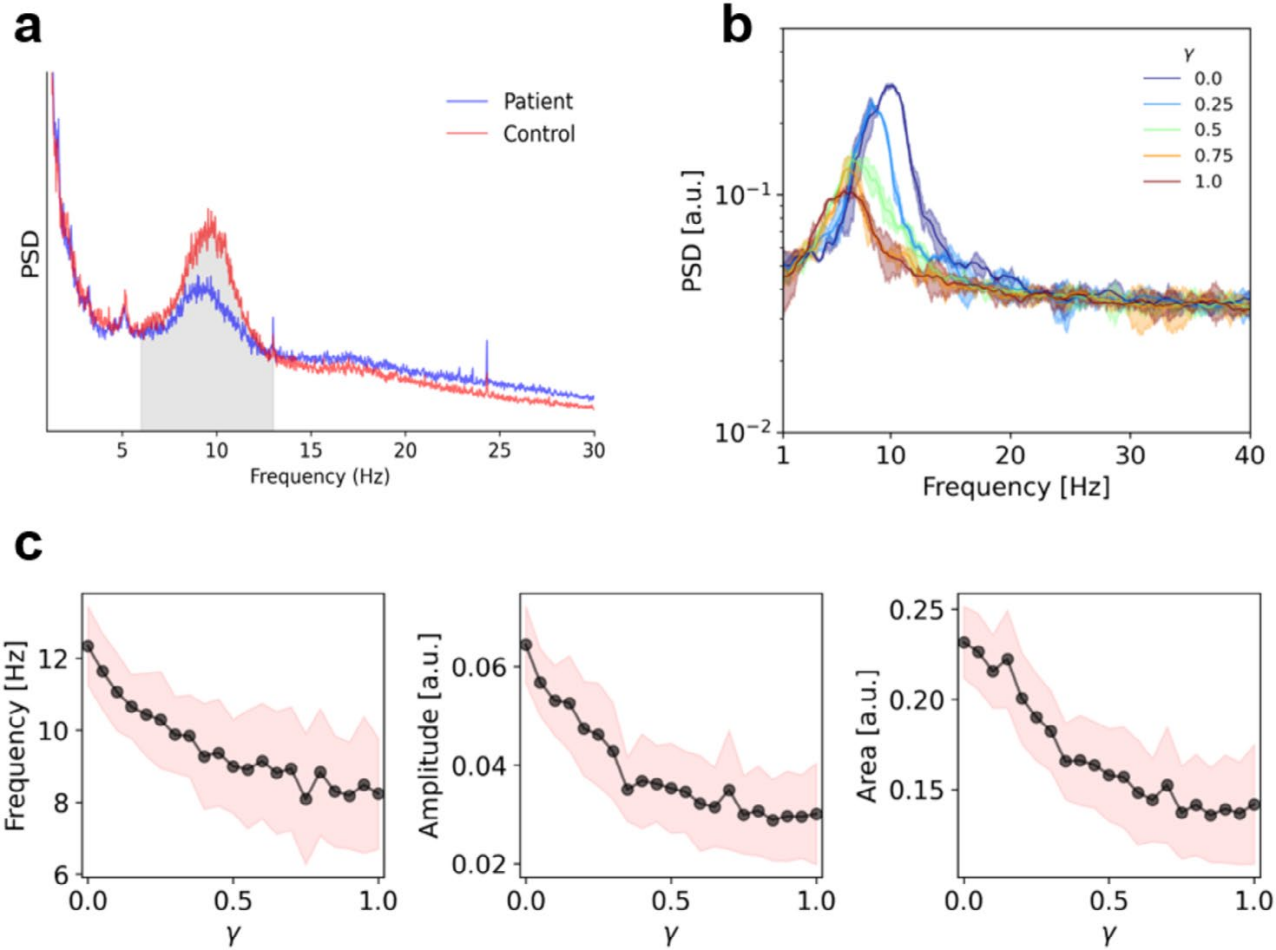
(a) Median Power Spectral Density (PSD) for both controls (in red) and MS patients (in blue). (b) Simulated PSD exhibits a peak in the alpha band, which decreases as the γ parameter increases. The full lines/shaded areas represent the average/standard deviations across different realizations for each value of gamma. (c) Peak frequency, amplitude of peak frequency, and area under the PSD as a function of γ. The confidence interval is represented by the average value ± the standard deviation for each frequency.

First of all, we confirmed the presence of a shift in the alpha frequency peak and a reduction in alpha band (8–13 Hz) power spectra in 18 MS patients compared to 20 controls (Figure 2a). Given that MS patients are more prone to fatigue, we have compared the theta/alpha ratio between groups (Cajochen et al. 1995; Cantero et al. 2002), which did not show statistically significant differences. As such, fatigue is unlikely to play a major role in our results. Then, we aimed to establish a relationship between the degree of lesion in specific tracts (which is expected to affect conduction velocities) and the power spectrum of MEG recordings. We will use the term “edge,” borrowing the terminology from network theory, to indicate the connections among brain regions. The topography of the lesions is reported in the Supporting Information. Specifically, we have binarized the lesion mask (i.e., at the edge‐level) and we have computed the corresponding nodal degree. Using this approach, the most affected lesions are understood as the ones upon which most lesioned edges are incident. This information is now reported in Figure S1, panel a and panel c. Finally, we report the sum of the binarized masks across subjects (Panel b).

Subject‐specific tractography was utilized to generate a large amount of synthetic data while varying the global coupling (scaling the structural connectivity matrix) and the parameter γ, that indicates the degree of influence of the lesions on the delay, from physiologically plausible ranges. Subsequently, low dimensional spectral features, including the area under the power spectral density (PSD), the amplitude of the peak frequency, and the peak frequency itself, were extracted from the synthetic dataset. Then we train neural density estimators to learn an invertible map between the effect of the lesions on conduction delays (represented by the parameter γ in our model) and the resulting data features extracted from random simulations. Note that, in each patient, the parameter γ weighs a different set of edges, that is, those who are lesioned in that patient. Figure 2b illustrates the power spectrum as a function of the frequency for various values of the parameter γ. This figure demonstrates that increasing the γ parameter, and thus assigning higher weights to lesions (resulting in greater impact on conduction delays) leads to more pronounced reduction in the amplitude of the alpha peak in the spectrum, and to a shift in its frequency. In Figure 2c, one can observe the changes in the amplitude and frequency of the peak, as well as the variations in the area under the PSD, calculated from the alpha spectrum as a function of γ. All these spectral features decrease as γ increases.

**FIGURE 3:**
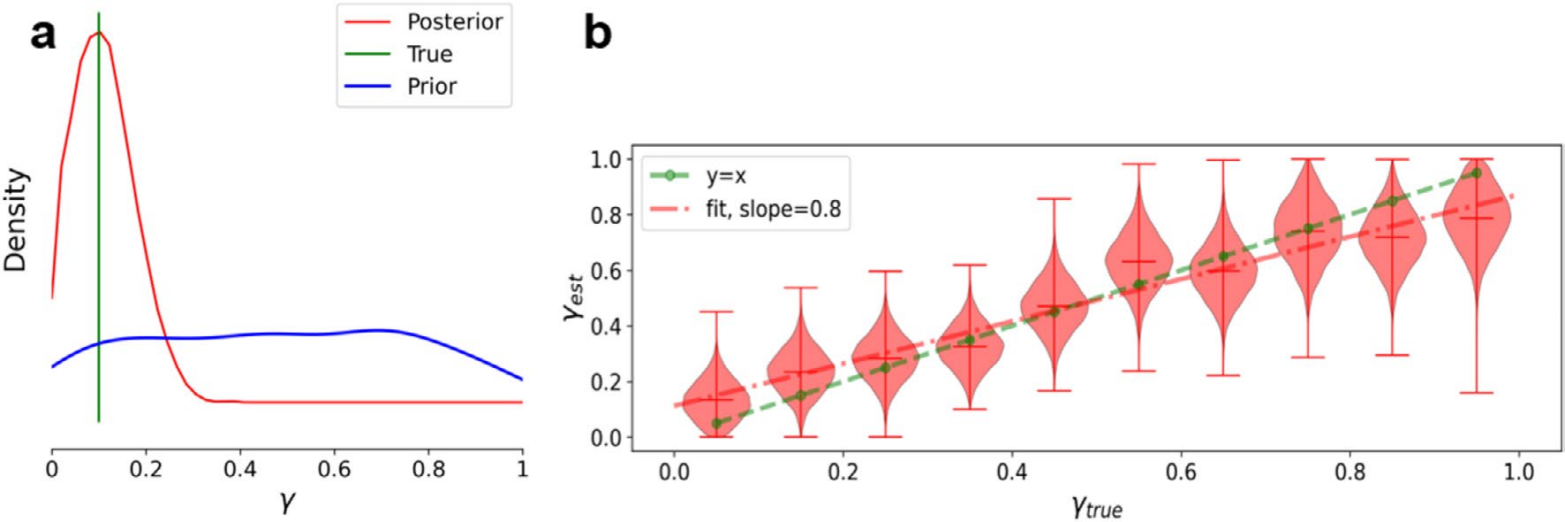
Validation of SBI using different model configurations over the parameter γ. (a) Posterior distribution in red, True parameter in green, and the uniform prior distribution in blue for a synthetic diagnostic of the model inversion pipeline. SBI provides accurate recovery of the parameter γ. (b) The violin plots in red show the estimated posterior densities by varying the parameter γ, the dashed red line represents a linear regression on the maximum values of the estimated posteriors; dashed green line represents the perfect fit (ground truth values). These results show the accuracy and consistency of the model inversion for different model configurations.

Then, we used SBI to build an invertible map between the values of γ (and G, i.e., the scaling factor for the connectome) to the corresponding data features. The validation of the SBI pipeline is shown in Figure 3. The neural density estimators (here, “Masked Autoregressive Flows”, Papamakarios et al. 2017) are trained using 5000 random simulations. Figure 3a shows the posterior distribution of γ given PSD features, while the parameters are drawn from a uniform prior distribution over the range [0,1]. For different model configurations, the SBI accurately estimates the posterior of parameter γ using PSD features. In Figure 3b, we showed different scenarios for γ, and we observed the accuracy of the estimated parameters versus the true values (with slope of 0.8 for the fitted maximum posteriors versus the true value). We note that for small/large values of γ there is a trend to over/under estimate the true values.

**FIGURE 4:**
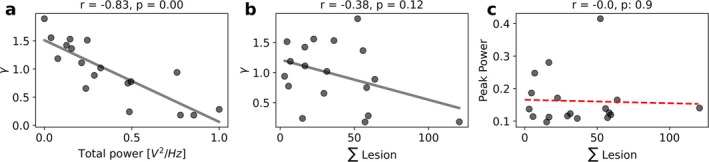
(a) Correlation between γ and the power (normalized across subjects between 0 and 1) in the alpha band (*r* = −0.83, *p* = 0.00). (b) Correlation between γ and the lesion load (*r* = −0.38, *p* = 0.12). (c) Correlation between the empirical peak and the lesion load (*r* = 0.00, *p* = 0.9).

We invert this map and use it to estimate the most likely γ, in each individual subject, given the empirically observed PSD features. We show that the most plausible γ values for the subjects relate, as expected, to the power in the alpha band (Figure 4a) (*r* = −0.83, *p* = 0.00). However, the lesion load itself does not relate to the amplitude of the alpha‐peak nor to the γ parameter (*r* = 0.00, *p* = 0.9, *r* = −0.38, *p* = 0.12, respectively), showing that the weighting of the lesions, that is, the effect that they exert on the slowing of conduction velocities, is likely topography‐dependent and, as such, patient specific.

Then we assessed the validity of the inferred individual γ values in predicting clinical disability. Using a multilinear model, we predicted clinical disability measured by the Expanded Disability Status Scale (EDSS), considering variables such as age, disease duration, total lesion volume, and inferred γ values as predictors. We have used R^2^ as a measure of the model's goodness‐of‐fit. Adding γ to the predictive model enhances the predictive power on clinical disability, although without reaching statistical significance, as shown in Supporting Information Figure S2. The model has been validated using a leave‐one‐out cross‐validation scheme. Moreover, it is evident that the total lesion load has less predictive power. Although failing to reach statistical significance, the increase in the explained variance implies clinical relevance despite being non‐predictive.

## 3 Discussion

The main goal of this work is to estimate a relationship between the lesions specific to each patient and the resulting conduction delays, via the changes in the power spectrum, particularly in the alpha band. We found a negative correlation between γ, a parameter that weighs the influence of the lesion mask, and the amplitude of the alpha peak in the spectrum. However, we also observe that the power peaks themselves do not correlate with the individual total lesion volume. In MS, the patients consistently exhibit a decrease in power within the alpha frequency band (8–13 Hz), as reported by several studies. Cover et al. (Cover et al. 2006) discovered a notable reduction in interhemispheric synchronization in the alpha band, measured via MEG among MS patients, while Bsar‐Eroglu (Bsar‐Eroglu et al. 1993) observed significant reductions in both alpha band synchronization and amplitude. These findings were further supported by Schependom (Van Schependom et al. 2021), who linked the decrease in alpha power to increased brain atrophy and lesion load. Additionally, Sorrentino et al. (Sorrentino et al. 2023) identified substantial differences in alpha band features between MS patients and age‐matched controls. Specifically, a shift in the power spectra of brain activity has been observed in MS, characterized by reduced power in the alpha frequency band and a shift in the peak frequency towards lower frequencies. This reduction in alpha power is believed to result from attacks by the immune system on the myelin, leading to a slowing down of nerve signal conduction, as evidenced by recent studies showing widespread delays throughout the brain in MS patients compared to healthy individuals (Berman et al. 2020; Sorrentino et al. 2022).

However, we also observe that the power peaks themselves do not correlate with the individual total lesion volume. Taken together, this finding highlights the importance of the specific topography of lesions (Gollo et al. 2018), since the impact of similar total lesion volumes on the overall spectral features varies greatly across patients. An interesting hypothesis would be that each tract holds a specific way to impact the overall large‐scale dynamics. In this case, lesions involving specific structures, such as the cortico‐thalamic loop or the posterior areas, might more prominently affect the global dynamics. Along these lines, it was shown that thalamic atrophy in MS is linked to widespread disruption of cortical functional networks, which is associated with poorer cognitive and clinical outcomes in MS patients. To test this hypothesis, we plan to investigate whether specific types of edge structural lesions affect conduction velocities in an edge‐specific fashion, or if each edge presents a unique contribution. To this end, larger multimodal datasets are needed. This hypothesis, besides the neuroscientific interest, also holds practical relevance. In fact, inferring edge‐specific delays is not feasible due to a parameter explosion. However, as we have seen, the specific topography of the lesions, that is, the specific edges that are damaged, plays a role in the final effect on the large‐scale dynamics. In this context, our work provides a way to translate patient‐specific lesion topographies to changes in the conduction velocities in all the edges involved, according to one rule. This way, we hope to lead to better personalization of large‐scale models, while preventing this problem from becoming too high‐dimensional.

Our study incorporates simulation‐based inference (SBI), a powerful tool for estimating latent parameters that cannot be measured directly in clinical settings. SBI lends itself nicely to several clinical applications, particularly for the early detection of neurological diseases. By leveraging patient‐specific data, SBI facilitates the personalization of computational models. For example, Hashemi et al. (2024) developed SBI for virtual brain models to investigate brain disorders, demonstrating that SBI improves inference precision and enhances biological plausibility. Such advancements contribute to precision medicine, enabling fast and reliable estimations of pathophysiological processes in a patient‐specific fashion. Furthermore, Hashemi et al. (2023) introduced the simulation‐based inference for the virtual epileptic patient, employing deep neural density estimators to perform amortized Bayesian inference on whole‐brain epilepsy models. In the context of our study, SBI is particularly important for translating patient‐specific topographies of lesions into changes in conduction velocities across all the affected edges in the brain network. SBI enables us to infer rules that govern the impact of lesions on delays while avoiding edge‐wise inference (which would be untreatable). Instead of directly working with time series data, SBI operates on data features extracted from simulations, treating the model as a black box that takes parameters as input and produces data features as output. By running many random simulations, SBI maps parameter values to data features, enabling inference by comparing these features with empirical data. This makes it a powerful tool for estimating latent parameters that are difficult to measure directly in clinical settings.

Hence, this approach enhances the personalization of large‐scale brain models, making them more adherent to the condition of each patient. For MS, this means providing a link from lesion patterns over the large‐scale and whole‐brain dynamics. As such, our work lays the basis for monitoring subclinical damage more effectively potentially leading to prompt clinical decisions (i.e., therapeutic changes). It is important to note that, while our model focuses on time delays, MS is a multifactorial disease, where myelin damage plays a central role, but other mechanisms, such as degenerative processes, also contribute significantly. Our model captures only a part of the disease's complexity. One key pathophysiological mechanism in MS involves delays in signal conduction due to myelin degradation. This results in slower nerve signal transmission. However, our model does not capture other aspects directly linked to neurodegeneration, some of which might also cause α and slowing (Abela et al. 2018; Corlier et al. 2019; Garcés et al. 2013; Ippolito et al. 2022). Despite these limitations, our model is meant as a tool for investigating the pathophysiology of MS. It effectively highlights the role of white‐matter lesions and their impact on neural conduction, which is a central feature of MS.

Furthermore, there are inherent limitations in the validation of the model's predictions on empirical data, due to volume conduction. In fact, the source‐reconstruction procedure only partially disentangles the field spread (i.e., the same source contributing to the activities recorded by multiple sensors) (Palva et al. 2018; Schoffelen and Gross 2011). In this sense, part of the zero‐lag components of the correlations observed in the empirical data might be spurious, which introduces some ambiguity in the interpretation of the fit of the simulated data to the empirical ones.

Finally, we should mention that, while the γ parameter captures conduction delay alterations linked to myelin pathology, providing a mechanistically relevant view of MS‐related neurophysiological changes, the peak frequency is computationally simpler. However, the personalized model enables hypothesis generation on individual damage impact and could predict functional effects of potential lesions, though validation through longitudinal studies is needed. In conclusion, developing and refining increasingly personalized models to monitor the therapeutic treatment of MS patients is crucial. The variability among individuals significantly influences treatment outcomes, and tailoring healthcare options to each patient's specific needs is expected to enhance treatment outcomes (Jirsa et al. 2017, 2023; Lang et al. 2023; Wang et al. 2023). This is the first study demonstrating the topography‐specific effect of myelin lesions on conduction delays, adding one step further to the personalization of models in individuals with MS.

## 4 Methods

### 4.1 Participants

Participants (both controls and patients) were recruited from the outpatient clinic of the Institute for Diagnosis and Cure Hermitage Capodimonte in Naples, Italy. Selection of patients with multiple sclerosis (MS) followed the 2017 McDonald criteria (Thompson et al. 2018), which excluded individuals under the age of 18, those with recent relapses or steroid treatments within three months, and those with a history of illicit drug, stimulant, amphetamine, barbiturate, and cannabinoid use or any other history of central nervous system disorders besides MS. Each patient underwent a comprehensive neurological examination, along with various assessments including the Expanded Disability Status Scale (EDSS). Table 1 provides a summary of the demographic data, key clinical characteristics, and primary radiologic findings for the MS cohort. Controls were chosen from caregivers and patients' partners. Ethical approval was obtained from the local Ethics Committee (Prot.n.93C.E./Reg. n.14‐17OSS).

**TABLE 1:** MS cohort features.

	Controls	MS	*p*
Age (years)	45.8 (±11)	44.9 (±9.9)	0.8
Education (years)	13.6 (±3.8)	13.8 (±5)	0.9
Gender (m/f)	6/14	6/12	0.3
Disease duration (months)	—	187.7 (±131.8)	—
EDSS	—	4.5 (±1.9)	—
SDMT	—	40.3 (±13)	—
FSS	—	36.1 (±14)	—
BDI	—	12.8 (±1.3)	—
LL	—	12.959 (±12.253) mm^3^	—

Abbreviations: DSS, Expanded Disability Status Scale; SDMT, Symbol Digit Modalities; FSS, Fatigue Severity Scale; BDI, Beck Depression Inventory; LL, lesion load.

### 4.2 MRI Acquisition and Processing

The MRI data acquisition and processing followed the methods described in Sorrentino et al. (Sorrentino et al. 2022). An MRI scanner operating at 1.5 Tesla (Signa, GE Healthcare) was utilized, with echo‐planar imaging employed for DTI (Diffusion Tensor Imaging) reconstruction and 3D‐FLAIR volume utilized for WM (White Matter) lesion segmentation. Preprocessing of the diffusion MRI data included correction for head movements and eddy current distortions, using software modules provided in the FMRIB software library (FSL; http://fsl.fmrib.ox.ac.uk/fsl). This step is crucial to ensure proper alignment and artifact‐free data, mitigating disturbances caused by head movement or other sources during scanning. Additionally, a brain mask was generated from the B0 images using the Brain Extraction Tool routine.

Subsequently, a diffusion tensor model was fitted to each voxel, allowing quantification of water diffusion direction and anisotropy in biological tissues. Using the Fiber Assignment by Continuous Tracking (FACT) algorithm, fiber tracts representing major connectivity pathways in the brain were generated. Two cortical study‐specific ROI datasets were obtained by masking ROIs available in the Desikan–Killiany–Tourville (DKT) atlas. Spatial normalization of each participant's FA volume was performed to obtain patient‐specific ROI sets.

Furthermore, MS lesion maps were generated by segmenting the 3D‐FLAIR volume using the lesion prediction algorithm implemented in the Lesion Segmentation Tool (LST toolbox version 3.0.0; www.statistical‐modeling.de/lst.html) for the Statistical Parametric Mapping software package (SPM). After obtaining the lesion map, the 3D‐FLAIR volume was aligned (or “coregistered”) with another type of MRI sequence called EPI (Echo Planar Imaging) for the same patient. This coregistration process, described by Ashburner and Friston in 1997 (Ashburner and Friston 1997), ensures that the images from different MRI sequences are spatially aligned with each other. Once the coregistration was completed, the transformation matrix used to align the 3D‐FLAIR volume with the EPI sequence was applied to the MS lesion volume. This step effectively transferred the spatial information of the lesions onto the same coordinate space as the EPI sequence. To ensure accurate alignment, the WM lesion volume was resampled using a method called nearest‐neighbor interpolation. This interpolation method involves assigning each new voxel in the resampled volume the value of the nearest voxel in the original volume, helping to preserve the integrity of the lesion information during the transformation process.

The resulting output of this process is a set of lesion masks specific to each patient, accurately aligned with the DTI volume. In this way, these lesion masks can be used for further analysis and investigation of the relationship between MS lesions and brain connectivity patterns obtained from DTI data.

Finally, the average length of fibers connecting each pair of ROIs and whether the voxels traversed by these fibers contained MS lesions were computed for the DKT ROI sets, utilizing an in‐house routine written in Interactive Data Language (IDL). The length of each tract was calculated for each subject by averaging the physical distances covered by the fibers within the tract. These distances were obtained by summing the straight‐line distances between the points where the fiber changes direction.

### 4.3 MEG Pre‐Processing and Source Reconstruction

Each participant underwent MEG recordings consisting of two eyes‐closed resting‐state segments, each lasting 3 min and 30 s. Electrooculogram (EOG) and electrocardiogram (ECG) signals were recorded to identify physiological artifacts such as eye blinks and heart activity. An anti‐aliasing filter was applied to the MEG signals, acquired at 1024 Hz, assuring that the signal is properly sampled without distortion or loss of information. A fourth‐order Butterworth IIR band‐pass filter (0.5–48 Hz) was subsequently applied. MEG preprocessing and source reconstruction procedures followed the detailed protocols outlined in (Sorrentino et al. 2021). Preprocessing and source reconstruction operations made use of the Fieldtrip Toolbox. Principal component analysis and independent component analysis were employed to eliminate environmental noise and physiological artifacts. Source reconstruction followed the protocols described in (Sorrentino et al. 2022).

In summary, the signal time series were reconstructed using 84 Regions of Interest (ROIs) based on the DKT atlas. Utilizing the volume conduction model proposed by Nolte (Nolte 2003), the reconstruction process involved applying the linearly constrained minimum variance (LCMV Van Veen and Buckley 1988) beamformer to reconstruct the signal sources based on the centroids of each ROI.

### 4.4 Design of the Synthetic Model

Whole‐brain models are computational representations designed to mimic the intricate workings of the entire human brain by integrating its various regions as a network node, placing a dynamical model at each brain region. These models strive to simulate the brain as a unified entity, considering both its anatomical structure, the nonlinear dynamics at each brain region, and the interactions among different brain regions. Their main objective is to deepen our understanding of how the brain processes information, generates consciousness, and performs cognitive functions, and notably dysfunctions at various brain diseases (Jirsa et al. 2023; Wang et al. 2023). Over time, whole‐brain models have proven invaluable in elucidating neuropathologies such as Alzheimer's disease, schizophrenia, epilepsy, traumatic brain injury, and others (Pathak et al. 2022; Wang et al. 2024). Their clinical utility extends to providing prognostic tools and predictive insights for various neurological disorders. However, given the diverse origins and mechanisms of brain pathologies, these models are customized to suit the specific etiology of each disease.

To effectively capture the dynamics of our system and construct a realistic brain network model with accurate connectivity and temporal delays, we opted to represent each ROI using a Stuart‐Landau oscillator (Cabral et al. 2022). The Stuart‐Landau model offers simplicity and versatility, making it suitable for studying a wide range of dynamic systems, particularly those with temporal delays. By incorporating a term for temporal delay into Stuart‐Landau's differential equation, this model provides a robust mathematical framework for analyzing the impact of delays on system dynamics. In contrast to other models, the Stuart‐Landau model explicitly accounts for temporal delays, enabling a more comprehensive understanding of their influence on overall system behavior over time.

The Stuart‐Landau system exhibits two potential solutions: damped or limit cycle solutions, contingent upon the bifurcation parameter a. For a < 0, we will have damped oscillations, while for a > 0, limit cycle solutions. In this study, we used a=−5 to reflect the dampened characteristics of local oscillations. As previously done in Sorrentino et al. (Sorrentino et al. 2023), oscillators within the system are interconnected via white matter, with coupling strength determined by subject‐specific DTI fiber counts ($c_{\mathrm{jk}}$). The activity of each ROI is expressed as:

(1) $$\dot{Z_{j}}=Z_{j}\left(a+i\omega_{j}-{\left|Z_{j}\right|}^{2}\right)+G\sum_{j\neq k}^{N}c_{\mathrm{jk}}\;\left[Z_{k}\;\left(t-\tau_{\mathrm{jk}}\right)-Z_{j}\left(t\right)\right]+\beta \;\left(\eta_{1}^{j}+i\eta_{2}^{j}\right)$$

where:

(2) $$\tau_{\mathrm{jk}}=\frac{d_{\mathrm{jk}}}{v_{\mathrm{jk}}}+\gamma \cdot l_{\mathrm{jk}}$$

Each ROI functions as a local E‐I (excitatory‐inhibitory) unit oscillating in the gamma band, set at 40 Hz ($\omega_{\mathrm{jk}}$).

Parameter G is the global coupling parameter that represents the overall level of interconnection between the different parts of the system, influencing the strength of interactions among diverse brain regions.

$\tau_{\mathrm{jk}}$ is the signal conduction time estimated by dividing inter‐node Euclidean distances $d_{\mathrm{jk}}$ by the conduction velocity $v_{\mathrm{jk}}$ plus a contribution caused by the lesion. In this case, $\tau_{\mathrm{jk}}$ depends on the degree of the lesion in every specific tract of the individual patient. $l_{\mathrm{jk}}$ represents the percentage of voxels within each fiber bundle, connecting a given pair of ROIs from the DKT atlas, that fall within a lesion as determined by the LST (Lesion Segmentation Tool). For each connection in the network, ljk quantifies the fraction of the tract's voxels that have been affected by lesions, providing a detailed measure of edge‐specific lesion severity and linking the model directly to the underlying physiological data. In other words, the delay (specific for every tract) depends on $l_{\mathrm{jk}}$ that describes how damaged the tract in question is, while γ is a parameter representing the intensity of the influence of the lesions on the delay. Random noise β (mean = 0, std. = 1e‐4) is introduced to each oscillator to mimic stochastic variations.

Note that macroscopic oscillations in the global order parameter Z near the α‐band (8–12 Hz) emerged despite individual units having a natural frequency of 40 Hz. This arises from the interplay of synchronization, time delays, and the brain's network structure. The model uses delayed differential equations to account for finite signal transmission speeds, causing phase lags that enable lower‐frequency collective oscillations (e.g., α‐band). Coupled with delays, oscillators synchronize metastability, transiently locking into coherent states and generating macroscopic oscillations distinct from individual frequencies. The brain's connectome amplifies specific frequency modes–such as the α‐band through resonance effects and excitatory–inhibitory interactions. Thus, α‐band oscillations in Z emerge from synchronization dynamics shaped by time delays and network architecture, shifting macroscopic behavior away from individual frequencies. In Supporting Information Figure S3, we report an example of empirical and simulated time series (and corresponding PSD) for a randomly selected patient and control.

### 4.5 Model Inversion

Inference is the top‐down method of drawing conclusions or making predictions based on observations. In other words, it is the process of advancing from initial premises and observations to logical conclusions by quantifying the uncertainty in a model, providing an objectification of the validity of the decision (Jirsa et al. 2023).

Bayesian approach provides the posterior probability of an event, given a set of observed data. Bayes theorem states that $p\left(\theta /y\right)=\frac{p\left(\theta \right)p\left(y/\theta \right)}{p\left(y\right)}$, where $p\left(\theta /y\right)$ is the probability of a hypothesis given the data (known as the posterior probability), $p\left(\theta \right)$ is the probability of the parameter before seeing any data (the probability of the hypothesis itself, known as the prior probability), $p\left(y/\theta \right)$ is the probability of data given the hypothesis (known as the likelihood) and p(y) is the probability of the data independently from any parameter (known as model evidence). This means that we can update our prior knowledge, represented by prior probabilities with new information provided by observed data to refine our estimates about parameters of interest, incorporating in this way both background knowledge and new empirical evidence to obtain a more accurate estimation of quantities of interest. In this study, the unknown parameter set is $\theta =\left\{\gamma ,G\right\}$, where γ is the weight of the lesion load in a specific patient and G is the coupling scaling factor, whereas y represents the PSD features extracted from the MEG data of each patient.

Efficient Bayesian inference faces challenges in evaluating the likelihood function ($p\left(y/\theta \right)$) given samples from the prior distribution. Particularly for whole‐brain scales, computational costs make likelihood‐based approaches sampling impractical. Simulation‐based Inference (SBI; Cranmer et al. 2020), or likelihood‐free inference, offers a solution by using low‐dimensional data features, efficiently performing Bayesian inference for high‐dimensional and complex models where calculating the likelihood function is intractable. In this case, the simulator can be used as a black‐box whose internal workings do not accessible and are not required to be differentiable, but it can generate synthetic data similar to the observed data (Hashemi et al. 2023). To efficiently conduct SBI, a class of deep artificial neural networks called Normalizing‐Flows (Rezende and Mohamed 2015) can be used to learn an invertible transformation between parameters and data features from a set of simulations with parameters drawn from prior distribution.

We first validate SBI on the whole‐brain model of Stuart‐Landau oscillators using synthetic data generated with subject‐specific structural connectomes by recovering ground‐truth parameters, then apply it to empirical MEG data. For the training (here using Masked Autoregressive Flows; (Papamakarios et al. 2017)), the parameters are drawn from a uniform prior in the range $\gamma \in U\left(0,1\right)$, with the budget of 5000 simulations. The set of low dimensional data features consists of the frequency, the peak amplitude of the mediated power spectrum, and the area under the curve in alpha band.

## Conflicts of Interest

The authors declare no conflicts of interest.

## Supporting information


**FIGURE S1.** Detailed anatomical Ljk networks. (a) Number of lesioned tracts per region; (b) Percentage of lesion for each tract; (c) Target regions most affected by lesions.

**FIGURE S2.** Clinical outcome prediction. (a–d) Variance explained by the model adding 4 predictors: age, duration, lesion load and estimated γ. The parameter γ enhances prediction accuracy in both classical multilinear (R^2^ = 0.2793; AdjR^2^ = 0.017226) (age β = 0.0227 ρ = 0.7328; duration β = 0.0015 ρ = 0.7547; lesion load β = −0.0000 ρ = 0.5921; γ β = −3.3099 ρ = 0.1366) and cross‐validated models (R^2^ = 0.29882; AdjR^2^ = 0.018348) (age β = 0.0327 ρ = 0.7229; duration β = 0.0011 ρ = 0.7421; lesion load β = 0.0000 ρ = 0.5968; γ β = −3.3972 ρ = 0.1784). (b–e) Predicted versus empirical EDSS scores; (c–f) Residuals evaluation.

**FIGURE S3.** Examples of time series and corresponding spectra, for a patient and a control, for empirical and simulated data. The figure displays the time series for both a control subject and a patient, along with their corresponding median power spectra. The observed data is shown in green, while the predicted values are depicted in purple. The estimated gamma value for the patient subject in this case is 0.71.

## Data Availability

The data that support the findings of this study are available on request from the corresponding author. The data are not publicly available due to privacy or ethical restrictions.
