# Supplementary figures and images for: Mapping Brain Lesions to Conduction Delays: The Next Step for Personalized Brain Models in Multiple Sclerosis
Source: Hum Brain Mapp. 2025 May 3;46(7):e70219. doi: 10.1002/hbm.70219 (PMC12048862; doi:10.1002/hbm.70219)

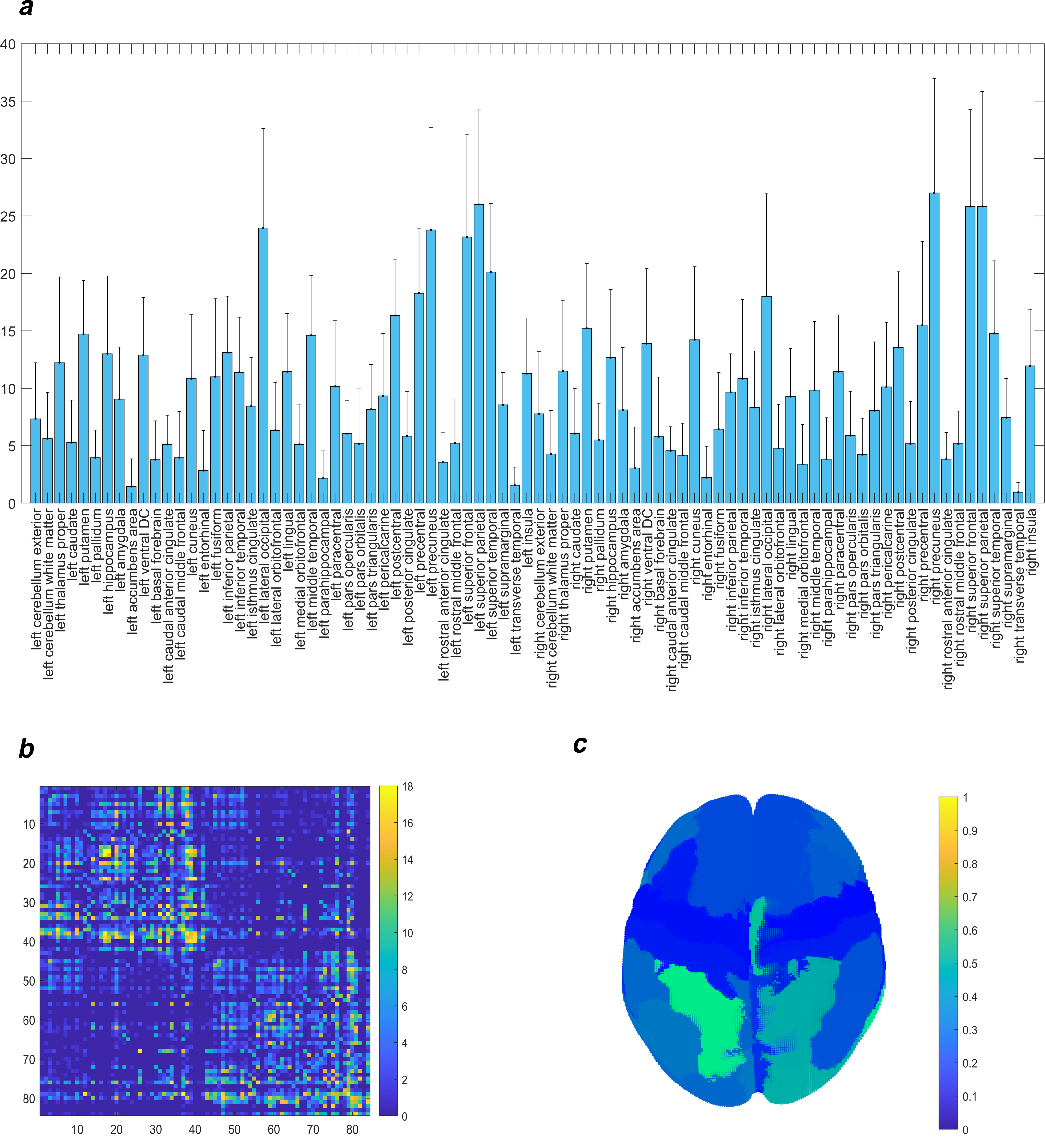

Supplement: Supplementary file 1 — FIGURE S1. Detailed anatomical Ljk networks. (a) Number of lesioned tracts per region; (b) Percentage of lesion for each tract; (c) Target regions most affected by lesions. [file HBM-46-e70219-s001.png]

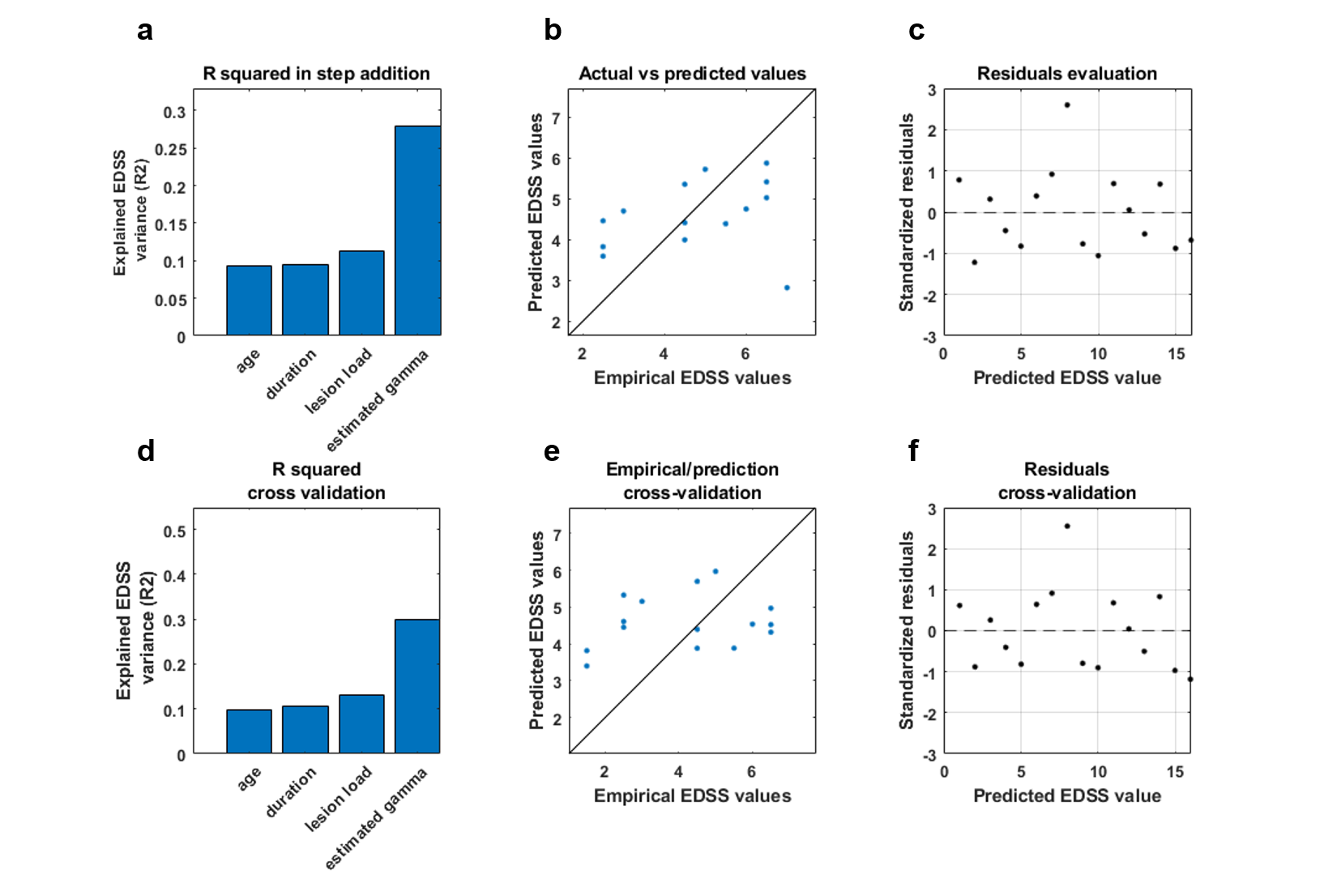

Supplement: Supplementary file 2 — FIGURE S2. Clinical outcome prediction. (a–d) Variance explained by the model adding 4 predictors: age, duration, lesion load and estimated γ. The parameter γ enhances prediction accuracy in both classical multilinear (R2 = 0.2793; AdjR2 = 0.017226) (age β = 0.0227 ρ = 0.7328; duration β = 0.0015 ρ = 0.7547; lesion load β = −0.0000 ρ = 0.5921; γ β = −3.3099 ρ = 0.1366) and cross‐validated models (R2 = 0.29882; AdjR2 = 0.018348) (age β = 0.0327 ρ = 0.7229; duration β = 0.0011 ρ = 0.7421; lesion load β = 0.0000 ρ = 0.5968; γ β = −3.3972 ρ = 0.1784). (b–e) Predicted versus empirical EDSS scores; (c–f) Residuals evaluation. [file HBM-46-e70219-s002.png]

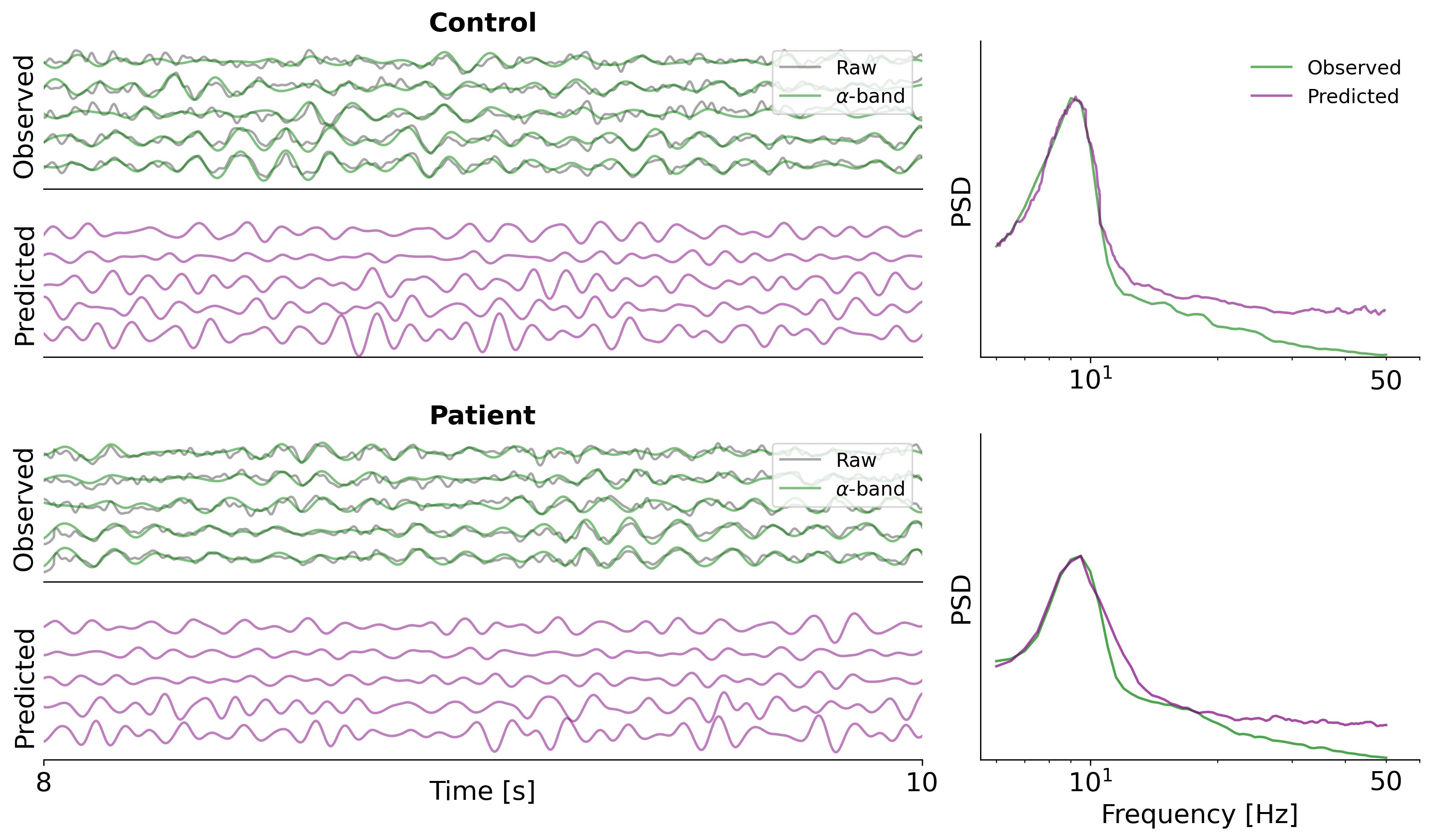

Supplement: Supplementary file 3 — FIGURE S3. Examples of time series and corresponding spectra, for a patient and a control, for empirical and simulated data. The figure displays the time series for both a control subject and a patient, along with their corresponding median power spectra. The observed data is shown in green, while the predicted values are depicted in purple. The estimated gamma value for the patient subject in this case is 0.71. [file HBM-46-e70219-s003.png]
